# Genomic characterization of individuals presenting extreme phenotypes of high and low risk to develop tobacco‐induced lung cancer

**DOI:** 10.1002/cam4.1500

**Published:** 2018-05-15

**Authors:** Juan Pablo Fusco, Guillermo Pita, María José Pajares, Maria Pilar Andueza, Ana Patiño‐García, Juan P. de‐Torres, Alfonso Gurpide, Javier Zulueta, Rosario Alonso, Nuria Alvarez, Ruben Pio, Ignacio Melero, Miguel F. Sanmamed, Maria Rodriguez Ruiz, Ignacio Gil‐Bazo, Jose María Lopez‐Picazo, Ciro Casanova, Rebeca Baz Davila, Antonio Agudo, Maria Dolores Lozano, Alvaro Gonzalez, Nuria Sala, Eva Ardanaz, Javier Benitez, Luis Montuenga, Anna Gonzalez‐Neira, Jose Luis Perez‐Gracia

**Affiliations:** ^1^ Department of Oncology Clinica Universidad de Navarra Pamplona Spain; ^2^ Health Research Institute of Navarra (IDISNA) Pamplona Spain; ^3^ Human Genetics Group Spanish National Cancer Centre (CNIO) Madrid Spain; ^4^ Program in Solid Tumors and Biomarkers Center for Applied Medical Research (CIMA) Pamplona Spain; ^5^ Centro de Investigación Biomédica en Red de Cáncer (CIBERONC) Spain; ^6^ Department of Pediatrics and Clinical Genetics Clinica Universidad de Navarra Pamplona Spain; ^7^ Pulmonary Department Clinica Universidad de Navarra Pamplona Spain; ^8^ Departments of Immunology and Oncology Clinica Universidad de Navarra and Center for Applied Medical Research (CIMA) Pamplona Spain; ^9^ Pulmonary Department and Research Department Hospital Universitario La Candelaria Santa Cruz de Tenerife Spain; ^10^ Research Unit Hospital Universitario La Candelaria Santa Cruz de Tenerife Spain; ^11^ Unit of Nutrition and Cancer, Cancer Epidemiology Research Program, Catalan Institute of Oncology‐ICO IDIBELL Barcelona Spain; ^12^ Translational Research Laboratory Catalan Institute of Oncology‐ICO IDIBELL Barcelona Spain; ^13^ Pathology Department Clinica Universidad de Navarra Pamplona Spain; ^14^ Department of Biochemistry Clinica Universidad de Navarra Pamplona Spain; ^15^ Navarra Public Health Institute CIBER Epidemiology and Public Health (CIBERESP) Pamplona Spain

**Keywords:** ATP10D, cancer risk, extreme phenotypes, genome‐wide association study, non‐small cell lung cancer, PDE10A, single nucleotide polymorphism, tobacco

## Abstract

Single nucleotide polymorphisms (SNPs) may modulate individual susceptibility to carcinogens. We designed a genome‐wide association study to characterize individuals presenting extreme phenotypes of high and low risk to develop tobacco‐induced non‐small cell lung cancer (NSCLC), and we validated our results. We hypothesized that this strategy would enrich the frequencies of the alleles that contribute to the observed traits. We genotyped 2.37 million SNPs in 95 extreme phenotype individuals, that is: heavy smokers that either developed NSCLC at an early age (extreme cases); or did not present NSCLC at an advanced age (extreme controls), selected from a discovery set (*n* = 3631). We validated significant SNPs in 133 additional subjects with extreme phenotypes selected from databases including >39,000 individuals. Two SNPs were validated: rs12660420 (*p*_combined _= 5.66 × 10^−5^; OR_combined _= 2.80), mapping to a noncoding transcript exon of *PDE10A*; and rs6835978 (*p*_combined _= 1.02 × 10^−4^; OR_combined _= 2.57), an intronic variant in *ATP10D*. We assessed the relevance of both proteins in early‐stage NSCLC. *PDE10A* and *ATP10D* mRNA expressions correlated with survival in 821 stage I–II NSCLC patients (*p* = 0.01 and *p* < 0.0001). PDE10A protein expression correlated with survival in 149 patients with stage I–II NSCLC (*p* = 0.002). In conclusion, we validated two variants associated with extreme phenotypes of high and low risk of developing tobacco‐induced NSCLC. Our findings may allow to identify individuals presenting high and low risk to develop tobacco‐induced NSCLC and to characterize molecular mechanisms of carcinogenesis and resistance to develop NSCLC.

## Introduction

In the recent years, genetic susceptibility to lung cancer has been explored by many genome‐wide association studies (GWAS), which have reported several genomic loci and candidate genes that exert moderate effects on lung cancer risk. The most well‐known loci and genes identified are the *CHRNA3* and *CHRNA5* subunits of the *α*7 nicotinic acetylcholine receptor (nAChR), located on the 15q25 region 1, 2, 3; *TERT* and *CLPTM1L* 4, 5, 6, 7, which are closely located at 5p15.33; *BAT3*, mapped to 6p21.23 8; and *GPC5* at 13q31.3 9. Most of these studies follow a random sampling design, including smoking habits as a covariate. Several noteworthy associations are found exclusively in subgroups of subjects defined upon their smoking habits. For example, the 15q25 locus is associated with lung cancer in former and current smokers, while the 13q31 locus is associated with susceptibility to lung cancer in never smokers 7, 9.

However, despite tobacco smoke being the most relevant known risk factor for lung cancer, no association studies have analyzed the individual risk that it confers through the study of subjects presenting extreme phenotypes of high and low risk to develop non‐small‐cell lung cancer (NSCLC) induced by tobacco. Such designs may help to identify high‐risk populations that could benefit from prevention and screening programs and to identify the molecular mechanisms that underlie such clinically relevant phenotypes.

We conducted a GWAS applying an extreme phenotype sampling in heavy smokers presenting high and low risk of developing NSCLC, and we validated our results using the same approach. We selected individuals who developed tobacco‐induced NSCLC at early onset and healthy subjects who did not develop NSCLC at an advanced age, despite having a long smoking history. We aimed to identify new susceptibility variants related to the respective extreme phenotypes.

## Patients and Methods

### Study design

We performed a two‐stage extreme phenotype study to increase the efficiency of discovering single nucleotide polymorphisms (SNPs) associated with the risk of developing tobacco‐induced NSCLC. We hypothesized that risk alleles would be strongly enriched in the phenotypic extremes, and therefore, a limited number of carefully selected individuals with extreme phenotypes might be sufficient to identify novel candidate genes and/or alleles 10.

**Figure 1:**
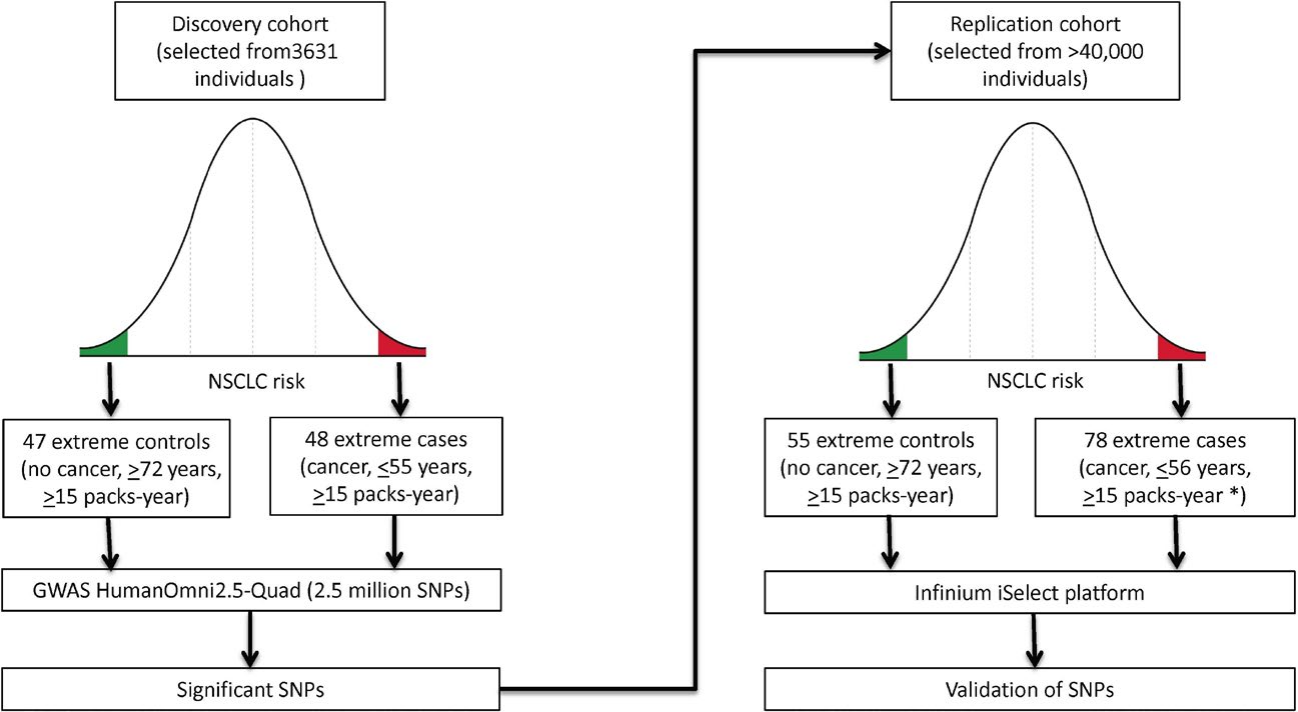
Study design. From our series, we selected the individuals presenting extreme phenotypes of high and low risk of developing NSCLC induced by tobacco. Heavy smokers that either developed NSCLC at an early age were selected as extreme cases, and individuals that did not develop NSCLC at an advanced age, despite heavy tobacco consumption, were selected as extreme controls. The specific thresholds to define these populations were set to select the most extreme phenotypes in our series. *We included selected cases that developed NSCLC at extremely young ages but presented tobacco consumption <15 pack‐years, given their phenotypic relevance and because we assumed that they were too young to achieve the threshold of smoke consumption.

We enrolled subjects into a discovery and a validation set (Fig. 1). The cancer cohort subjects (extreme cases) were selected from heavy smokers (≥15 packs‐years) with histologically confirmed diagnosis of NSCLC at an early age (≤55 years). In the validation series, we included selected cases that developed NSCLC at extremely young ages but presented tobacco consumption <15 packs‐years, given their phenotypic relevance and because we assumed that they were too young to achieve the threshold of smoke consumption. We also included some borderline cases for age (≤56 years). The cancer‐free cohort individuals (extreme controls) were selected from heavy smokers (≥15 packs‐years) that had not developed NSCLC or any other type of cancer at an advanced age (≥72 years). The thresholds for tobacco consumption and age were set with the aim to select from our series the individuals presenting the most extreme phenotypes regarding individual risk of developing NSCLC induced by tobacco.

The discovery set was recruited among 3631 patients included in the databases of the University Clinic of Navarra (Pamplona, Spain), Center for Applied Medical Research (CIMA, Pamplona, Spain), and Hospital Universitario Nuestra Señora de La Candelaria (Tenerife, Spain).

The independent validation set was recruited from the Spanish branch of the European Prospective Investigation in Cancer (EPIC) Project (http://www.epic-spain.com), which includes genomic DNA samples and clinical data from 39,880 individuals, and from additional new cases from the University Clinic of Navarra.

Samples and data from patients were processed following standard operating procedures approved by the respective Ethical and Scientific Committees. All patients gave written informed consent to allow the use of their biological samples and clinical data for research purposes. The study protocol was approved by the Ethics Committee of the University Clinic of Navarra.

### DNA genotyping

Genomic DNA was obtained from peripheral blood mononuclear cells using the QIAamp DNA Mini Kit (Qiagen Iberia, Madrid, Spain) according to the manufacturer's instructions and stored at −20°C until use. Genotyping in the discovery set was performed using the Illumina HumanOmni2.5‐Quad BeadChip according to the manufacturer's protocols (Illumina, San Diego, CA, USA). Genotyping in the validation set was performed using the Infinium assay following the manufacturer's instructions (Illumina).

### Statistical analysis

Discovery and validation analyses, per‐allele odds ratios (OR), and standard errors were estimated for each SNP using a multivariate logistic regression model, adjusting for sex. The covariates age and tobacco consumption were used for the design, and therefore, they were not included in the statistical analysis.

Genotype clustering and calling were carried out using GenTrain v2.0 in GenomeStudio v2011.1 (Illumina). Samples were excluded from further analyses if they had more than 2% missing genotype data SNPs with a call rate<0.95 or deviated from the Hardy–Weinberg equilibrium (HWE) (*p*‐value ≤1 × 10^−5^) were also excluded. From the discovery analysis, SNPs with a *p*‐value ≤5 × 10^−4^ and minor allele frequency (MAF) > 5% in the single SNP analysis were selected for validation. When two SNPs presented a very high correlation (*r* ^2^ > 0.8), they were considered redundant, and only the one that presented the highest Illumina score was selected.

### Genomic imputation

To find map causing variants, data imputation for the untyped SNPs at all the validated SNPs was performed using IMPUTE v2.3.2 11 for samples from the discovery set using phased haplotypes from the 1000 Genomes Phase 3 (b37) panel as reference 12. Those imputed genotypes with scores >0.3 were tested for association.

### Assessment of the prognostic value of mRNA expression and protein expression of target genes

We attempted to validate the functional relevance of the genes associated with the validated SNPs in NSCLC, assessing the expression of the related mRNA and proteins in patients with early‐stage NSCLC. We correlated the transcriptomic data of *ATP10D* and *PDE10A* with recurrence‐free survival (RFS) and overall survival (OS) using the KM‐Plotter application 13 in patients with early‐stage NSCLC. The database used (http://kmplot.com) includes gene expression data and survival information on 821 patients with stage I–II NSCLC, downloaded from the Cancer Biomedical Informatics Grid (CaBIG), the Gene Expression Omnibus (GEO), and the Cancer Genome Atlas (TCGA). Survival curves were estimated using Kaplan–Meier curves. Significant differences between groups were tested using the log‐rank test. RFS and OS were calculated respectively from the date of surgery to the date of recurrence or death.

We assessed the prognostic value of PDE10A protein expression in tumor samples from 149 patients with stage I–II NSCLC resected at University Clinic of Navarra. PDE10A immunohistochemical assay and staining scores established by semiquantitative analysis were performed as previously described 14. We used an anti‐human PDE10A antibody (Genetex) diluted 1:500. The specificity of PDE10A antibody was demonstrated by Western blot analysis and immunocytochemistry of cell lines expressing different levels of the protein. Isotype and negative (omission of the primary antibody) controls were performed 14.

The univariate and multivariate Cox proportional hazards modeling were used to determine the effects of variables on cancer‐specific OS and RFS. Variables with *p* < 0.1 in the univariate analysis were included in the multivariable analysis. The proportional hazards assumption was examined by testing interactions between the covariables of the final model and time. Statistical analysis was performed using SPSS 15.0 (Chicago, Ill., USA) and Stata12 (College Station, TX: USA).

## Results

### Identification and validation of SNPs related to individuals presenting extreme phenotypes of developing NSCLC induced by tobacco

We analyzed 228 individuals with extreme phenotypes, 95 in the discovery set and 133 in the validation set (Table 1). The discovery set included 47 extreme cases that developed NSCLC at an early age (mean: 49 years) and were heavy smokers (mean: 41 packs‐years). Extreme controls were 48 individuals that did not develop NSCLC at an advanced age (mean: 76 years) despite high tobacco consumption (mean: 69 packs‐years). One control from the discovery set was excluded due to low call rate (call rate <0.98). The validation set included 78 extreme cases (mean age: 49 years; and 38 packs‐years) and 55 extreme controls (mean age: 77 years; and 48 packs‐years).

**Table 1:** Characteristics of patients

	Discovery set (*n* = 95)		Validation set (*n* = 133)	
	Extreme cases	Extreme controls	Extreme cases	Extreme controls
*N*	47	48	78	55
Sex M/F	31/16	43/5	52/26	51/4
(%M/%F)	(66/34)	(89.4/10.6)	(66.7/33.3)	(92.7/7.3)
Mean age, years	49	76	49	77
(range)	(38–55)	(72–84)	(35–56)	(72–85)
Mean tobacco consumption,	41	69.4	38	48
packs‐year (range)	(15–99)	(40–150)	(4–101)	(16–123)
Histological subtypes				
Adenocarcinoma	29	NA	44	NA
Squamous cell carcinoma	14		12	
Other	4		21	

NA, not applicable.

We successfully genotyped 2,379,855 variants in the discovery set, and 61,061 were excluded from the analysis due to low genotyping call‐rate (0.95), and 370 were deviated from HWE in controls, leaving 2,318,553 for further analysis. Thirty‐six SNPs presenting high association in the GWAS (*p* < 5 × 10^−4^, Table S1) were analyzed in the validation set.

Two SNPs were successfully replicated in the validation set (Table 2). The strongest validated association was with SNP rs12660420 at the 6q27 locus (combined *p* = 5.66 × 10^−5^; combined OR = 2.80, 95% CI: 1.69–4.61), which maps to a non‐coding transcript of the *PDE10A* gene. This SNP tags a linkage disequilibrium (LD) block of 85.8Kb in chromosome 6 (coordinates 166,109,230–166,195,063) (NCBI build 37 assembly), as defined by the furthest upstream and downstream SNPs displaying a detectable correlation (*r* ^2^ > 0.20) with rs12660420.

**Table 2:** Single nucleotide polymorphisms validated in extreme cases versus extreme controls

SNPs	Chromosome	Position	References	Alternate	Study	*p*‐value	MAF	OR	95% CI
rs12660420	6	166193932	C	T	Discovery	1.48E‐04	0.28	5.29	2.24–12.51
					Validation	0.04805	0.32	1.88	1.01–3.5
					Combined	5.66E‐05	0.31	2.80	1.69–4.61
rs6835978	4	47500814	A	G	Discovery	3.69E‐04	0.24	5.15	2.09–12.7
					Validation	0.04303	0.32	1.80	1.02–3.19
					Combined	1.02E‐04	0.31	2.57	1.6–4.1

CI, confidence interval; MAF, minor allele frequency; OR, odds ratio; SNP, single nucleotide polymorphisms.

Associations between SNPs and NSCLC risk were assessed by logistic regression analyses adjusted for sex. Additive model of inheritance was considered. ORs are per copy of the specified minor allele. Chromosome positions are based on Genome Reference Consortium Human Build 37 (GRCh37/hg19).

**Figure 2:**
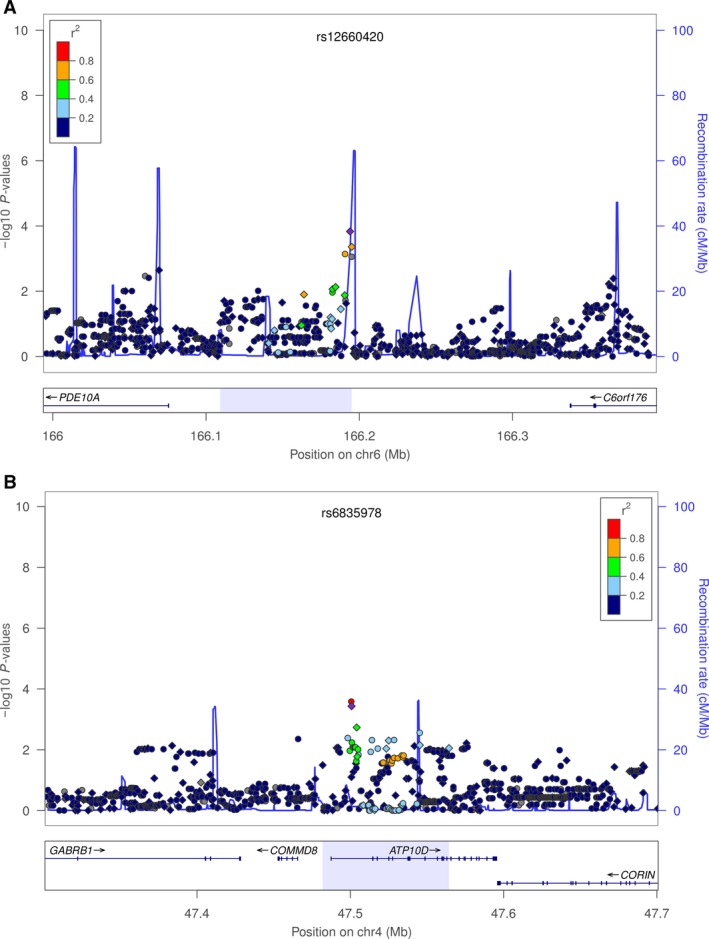
Association and recombination plot of the LD block containing the validated SNPs and ±200 kb boundaries. Plot shows the genomic region containing (A) rs12660420 (B) rs6835978 SNPs associated with lung cancer and the −log10 association *p*‐values of genotyped and imputed SNPs in the discovery set (MAF > 5%). Circles and diamonds indicate genotyped and imputed SNPs, respectively. Recombination rates are also shown. SNP color indicates the strength of LD (*r* ^2^) with rs12660420 (A) or rs6835978 (B). The rectangle below the plot shows the genes mapping in the region as well as, shaded in light blue, the LD block tagged by the associated SNPs. Recombination rates are based on the 1000 Genomes Project, and genomic coordinates are based on Genome Reference Consortium Human Build 37 (GRCh37/hg19). Plots were generated using LocusZoom software 31.

Subsequently, genotypes of all other known variants in the locus with minor allele frequency >5% were imputed to this locus. In total, 275 SNPs were reliably imputed (imputation *r* ^2^ score>0.3) and analyzed but none showed a lower *p*‐value than rs12660420. The LD plot showing the *p*‐values of these SNPs is shown in Figure 2. The related gene, *PDE10A*, encodes a protein that belongs to the cyclic nucleotide phosphodiesterase family. *PDE10A* expression correlates with lung cancer prognosis, and its pharmacological inhibition suppresses growth of NSCLC cell lines 15, so it seems a plausible candidate gene for lung cancer risk. Although the validated SNP lies in a non‐coding transcript of the *PDE10A* gene, genetic variation in regulatory elements may influence expression.

The second validated SNP was rs6835978 (chr4:47498797) in 4p12 (combined *p* = 1.02 × 10^−4^; combined OR = 2.57, 95% IC: 1.6–4.1, Table 2), an intronic variant in the *ATP10D* gene (ATPase phospholipid transporting 10D) 16. *ATP10D* has not previously been related to lung cancer or other tumors. rs6835978 tags to a LD block of 82Kb in chromosome 4 (47,481,971–47,564,368), defined as described above. A total of 228 SNPs were reliably imputed in the region, and rs6835978 remained the most significantly associated SNP (along with rs12510653, in complete LD with it [*r* ^2^ = 1]).

### Assessment of the prognostic value of mRNA expression and protein expression of target genes in patients with early‐stage NSCLC

**Figure 3:**
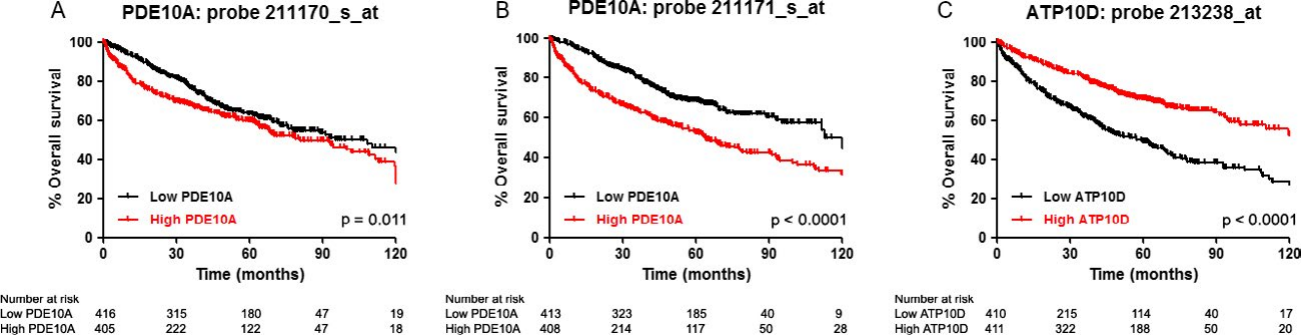
Correlation of the transcriptomic data of *PDE10A* an *ATP10D* with overall survival (OS) using the Kaplan–Meier Plotter application in several databases including 821 patients with stage I–II NSCLC. Panels A and B: OS according to PDE10A mRNA expression levels using probes 211170 (A) and 211171 (B). Panel C: OS according to ATP10D mRNA expression levels using probe 213238.

We evaluated the prognostic value of mRNA expression of *PDE10A* and *ATP10D* with the in silico tool KM‐plotter 13 in 821 patients presenting stage I–II NSCLC. We analyzed *PDE10A* (probes 211170 and 211171) and *ATP10D* (probe 213238). We classified patients into two groups according to the median value of expression of each biomarker. Patients with higher *PDE10A* mRNA expression showed decreased OS using both probes (*p* = 0.011 and *p* < 0.0001; Fig. 3, panels A and B). Low mRNA expression of *ATP10D* was associated with shorter OS (*p* < 0.0001; Fig. 3, panel C).

**Figure 4:**
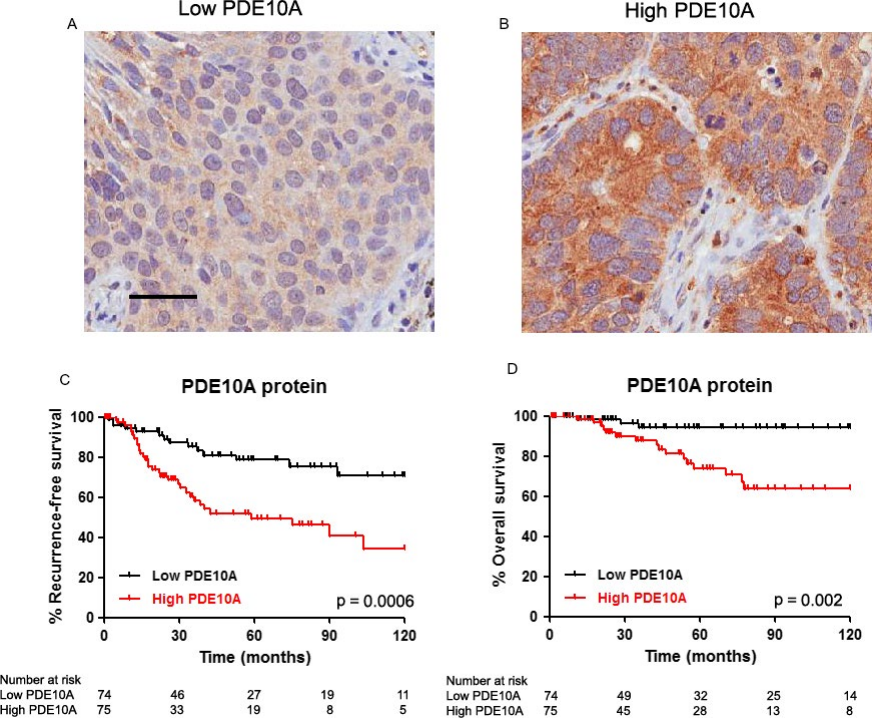
Correlation of PDE10A protein expression with overall survival (OS) and relapse‐free survival (RFS) in 149 patients with stage I–II NSCLC. Panels A and B: representative examples of tumors presenting low (A) and high (B) PDE10A staining. Scale bar: 50 micras. Panel C: RFS according to PDE10A staining. Panel D: OS according to PDE10A staining.

We assessed the prognostic value of PDE10A protein expression in 149 patients with stage I–II NSCLC (Table S2), followed during a median period of 46.5 months (interquartile range: 91.6–22.0). High tumor expression of PDE10A correlated with decreased RFS (*p* = 0.0006) and OS (*p* = 0.002) (Fig. 4). PDE10A expression correlated significantly with RFS and OS in the univariate analysis (Table 3). In the multivariate analysis, PDE10A expression was the strongest independent prognostic factor, over stage and age, and the only one that retained significance for both RFS (HR = 2.637; 95% CI = 1.381–5.036; *p* = 0.003) and OS (HR = 2.909; 95% CI = 1.376–6.144; *p* = 0.005).

**Table 3:** Univariate and multivariate Cox regression analyses of PDE10A protein expression for recurrence‐free survival (RFS) and overall survival (OS) in patients with stage I–II NSCLC

Factor	*n*	RFS				OS			
		Univariate HR (95% CI)	*p*	Multivariate HR (95% CI)^a^	*p*	Univariate HR (95% CI)	*p*	Multivariate HR (95% CI)^a^	*p*
PDE10A									
Low	74	–	–	–	–	–	–	–	–
High	75	2.831 (1.501–5.340)	0.001	2.637 (1.381–5.036)	0.003	2.86 (1.371–5.970)	0.005	2.909 (1.376–6.144)	0.005
Stage									
I	101	–	–	–	–	–	–	–	–
II	48	1.742 (0.963–3.151)	0.066	1.425 (0.779–2.607)	0.250	1.971 (1.011–3.840)	0.046	1.700 (0.864–3.331)	0.125
Age									
<65	74	–	–	–	–	–	–	–	–
>65	75	1.361 (0.755–2.457)	0.305	–	–	1.822 (0.927–3.579)	0.082	2.065 (1.042–4.091)	0.038

OS, overall survival; RFS, relapse‐free survival.

a Adjusted by gender, stage, and age.

## Discussion

We identified and validated two novel variants associated with individuals presenting extreme phenotypes of risk to develop NSCLC induced by tobacco. To our knowledge, this is the first study that uses this strategy to identify novel genetic variants related to NSCLC cancer risk. This methodology consists of studying reduced groups of individuals with very characteristic and clinically relevant phenotypes, thus enriching biomarker expression in the individuals studied, and it has allowed outstanding cancer biomarkers to be identified, as reviewed elsewhere 17. In the discovery phase, we used the HumanOmni2.5‐Quad BeadChip array, a high‐density chip that allows genotyping of 2.5 million SNPs across the whole genome, targeting genetic variation down to 1% MAF. To our knowledge, this is the first study that assesses cancer risk using this powerful platform.

The strongest validated association was SNP rs12660420, which maps to a non‐coding transcript of *PDE10A*. This gene encodes a protein that belongs to the cyclic nucleotide phosphodiesterase family. Phosphodiesterases (PDE) represent a large superfamily of hydrolases that control the intracellular levels of cyclic nucleotides by hydrolyzing cAMP and cGMP to 5_AMP and 5_GMP and are critical regulators of the myriad physiological and pathophysiological processes under cyclic nucleotide control 18. PDE4, a well‐known target for therapy of chronic obstructive pulmonary disease 19, is also expressed in lung cancer and promotes lung cancer progression 20. Preclinical data indicate that PDE4 inhibitors have also a role in the treatment for EGFR‐mutant NSCLC patients with high pretreatment levels of BIM and mTOR 21. Li et al. showed that PDE10A mRNA and protein levels are overexpressed in colon tumor cells as compared to normal colonocytes; that silencing *PDE10A* suppressed the growth of colon tumor cells; and that overexpression of *PDE10A* promotes growth of colonocytes and adenoma cells 22. The same group recently reported that Pf‐2545920, a highly specific *PDE10A* inhibitor, inhibits colon tumor cell growth at concentrations that increase cGMP and cAMP levels and activate PKG and PKA; and that Pf‐2545920 reduces *β*‐catenin‐mediated transcription of survivin, resulting in caspase activation and apoptosis 23. Shen et al. 15 have reported that *PDE10A* mutations are predicted to influence *PDE10A* allosteric regulation in lung adenocarcinoma; that high levels of *PDE10A* expression correlate with worsened lung adenocarcinoma prognosis; and that Pf‐2545920 also suppresses the growth of NSCLC cell lines. Finally, *PDE10A* somatic mutations have been described in up to 19% of prostate cancers and correlated with increased levels of phosphorylated C‐AMP responsive element binding protein (p‐CREB) 24. These data suggest that PDE10A might be a relevant target for cancer therapy and prevention.

The second validated variant, SNP rs6835978, is an intronic variant of *ATP10D*, which belongs to a subfamily of P‐type ATPases implicated in the translocation of phospholipids from the exoplasmic to the cytoplasmic leaflet of the cellular biological membranes. Although *ATP10D* has not previously been related to NSCLC, genetic variation in this gene has been associated with levels of plasma sphingolipids 25. Bioactive sphingolipids, such as sphingosine‐1‐phosphate (S1P), and ceramides, are signaling molecules involved in the activation of pathways that are directly relevant to carcinogenesis 26. Ceramide plays a major role in the development of chronic pulmonary diseases and has also been involved in lung cancer development induced by cigarette smoke 27. Alberg et al. reported that higher concentrations of S1P and total ceramide in plasma were associated with an increased future risk of lung cancer 28.

We analyzed the prognostic value of *PDE10A* and *ATP10D* mRNA and PDE10A protein levels in patients with stage I–II NSCLC. We confirmed that high and low mRNA expression, respectively, of *PDE10A* and *ATP10D* significantly correlated with shorter survival. To our knowledge, this is the first association of ATP10D with cancer prognosis. We confirmed that PDE10A protein levels in stage I–II NSCLC patients correlated with decreased survival. The results that we obtained for mRNA and protein expression of PDE10A are consistent among them and also with previous results reported in NSCLC 15. These results render *PDE10A* and *ATP10D* as potentially useful prognostic and therapeutic targets in NSCLC.

Tobacco‐induced NSCLC represents one of the most relevant challenges to public health. Therefore, the identification of genetic factors that confer to individuals either an increased risk or an intrinsic protection to develop the disease would be of critical relevance, allowing the identification of high‐risk populations, in which tobacco cessation and screening programs may be most beneficial. Most importantly, it may lead to further understanding of mechanisms of carcinogenesis and natural protection against cancer, which might inspire new therapeutic strategies. Interestingly, even though our study was specifically designed for NSCLC, the individuals in the cancer‐free cohort did not develop any other tumors. Therefore, our results may also relate to other neoplasms, especially those related to tobacco.

To our knowledge, this is the first study that defines and targets phenotypes of decreased risk to develop tobacco‐induced NSCLC and that attempts to characterize the genetic causes of such protection. The study of these proficient cancer risk phenotypes (PROCARPs) may allow to identify the underlying protective mechanisms and may provide valuable targets for cancer prevention. The identification of PROCARPs is challenging, because it requires to distinguish individuals presenting a truly decreased cancer risk from others which just present absence of the disease (i.e., they are non‐apparent phenotypes) 29. We hypothesized that the selection of individuals that are overexposed to either extrinsic or intrinsic cancer risk factors and yet do not develop cancer may allow to identify and study PROCARPs 17, 30. On the other hand, phenotypes of increased cancer risk (deficient cancer risk phenotypes, DECARPs) are apparent phenotypes that are readily identifiable (e.g., cancer familiar syndromes, cancer development at young ages, etc.) 29.

The main limitation of our study is that the sample size is limited due to the difficulty to enroll extreme individuals. This may impair to reach *p*‐values of genome‐wide significance. Nevertheless, to overcome this limitation, we explored mRNA and protein expression of *PDE10A* and *ATP10D* in two different patient populations of early‐stage NSCLC, and we found very significant correlations with survival. Functional validation will also be required to confirm the relevance of our findings. Also, despite our efforts to recruit highly selected individuals, phenotypic heterogeneity may persist regarding demographical and clinical characteristics. Nevertheless, this limitation constitutes in fact the greatest opportunity to improve this strategy, through the application of more stringent selection criteria to identify more homogeneous phenotypes with regard to age, tobacco consumption, or other relevant variables. Our strategy may also be pursued using different high‐throughput techniques, such as exome or genome sequencing or assessment of the transcriptome, and epigenome. Finally, additional studies will be required to further elucidate the optimal definitions of extreme phenotypes.

In summary, we identified and validated two new genetic variants in *PDE10A* and *ATP10D* associated with individuals presenting extreme phenotypes of high and low risk of developing tobacco‐induced NSCLC, and we confirmed the prognostic relevance of the associated proteins in early‐stage NSCLC. Our findings may have relevant implications to define high‐risk cancer populations and to characterize molecular mechanisms of carcinogenesis and resistance to develop cancer, thus supporting the feasibility of extreme phenotype selection studies in cancer research.

## Conflicts of Interest

The authors declare no conflicts of interest.

## Supporting information


**Table S1.** SNPs presenting high association in GWAS (*p* < 5 × 10^−4^).
**Table S2.** Clinicopathological features of 149 stage I–II NSCLC patients.

**Appendix S1.** Immunohistochemical analysis.
