# Supplementary material for: Genomic characterization of individuals presenting extreme phenotypes of high and low risk to develop tobacco‐induced lung cancer
Source: Cancer Med. 2018 May 15;7(7):3474–83. doi: 10.1002/cam4.1500 (PMC6051154; doi:10.1002/cam4.1500)
Supplement: Supplementary file 1 — Table S1. SNPs presenting high association in GWAS (p < 5 × 10^−4^). Table S2. Clinicopathological features of 149 stage I–II NSCLC patients. [file CAM4-7-3474-s001.docx]

| **Supplementary table 1: SNPs presenting high association in GWAS (p<5x10-4).** | | | | |  |
| --- | --- | --- | --- | --- | --- |
| **SNP ID** | **Chr** | **Position** | **Gen** | **MAF** | **P value** |
| rs1189107 | 14 | 56917786 | *37kb5' of C14orf101* | 0·30 | 4·46E-05 |
| rs1300661 | 14 | 56919803 | *35kb5' of C14orf101* | 0·30 | 7·41E-05 |
| rs2825830 | 21 | 21197762 | *68kb3' of AP000946.2* | 0·44 | 7·46E-05 |
| rs17405280 | 5 | 31384619 | *16kb3' of DROSHA* | 0·43 | 7·91E-05 |
| rs9983664 | 21 | 21091587 | *174kb3' of AP000946.2* | 0·45 | 9·22E-05 |
| rs2235967 | 14 | 68249499 | *ZFYVE26* | 0·33 | 1·18E-04 |
| rs4811157 | 20 | 49949839 | *44kb3' of AL079339.1* | 0·37 | 1·22E-04 |
| rs6727285 | 2 | 5488456 | *201kb3' of AC107057.1* | 0·39 | 1·32E-04 |
| rs12660420 | 6 | 166193932 | *PDE10A* | 0·28 | 1·48E-04 |
| rs12853031 | 23 | 6126513 | *NLGN4X* | 0·50 | 1·49E-04 |
| rs17064225 | 5 | 174417049 | *CTC-281M20.1* | 0·24 | 1·78E-04 |
| rs6693447 | 1 | 2330190 | *RER1* | 0·43 | 2·26E-04 |
| rs656661 | 5 | 31384205 | *16kb3' of DROSHA* | 0·40 | 2·30E-04 |
| rs73160755 | 12 | 132177959 | *18kb5' of SFSWAP* | 0·29 | 2·37E-04 |
| rs13049227 | 21 | 28097013 | *112kb3' of ADAMTS1* | 0·47 | 2·51E-04 |
| rs1225147 | 11 | 76555560 | *16kb5' of ACER3* | 0·20 | 2·53E-04 |
| rs12504035 | 4 | 131121249 | *176kb5' of RP11-521E5.1* | 0·17 | 2·65E-04 |
| rs11095019 | 23 | 6124767 | *NLGN4X* | 0·49 | 2·69E-04 |
| rs56845266 | 7 | 64081538 | *45kb5' of ZNF107* | 0·17 | 2·82E-04 |
| rs2306911 | 17 | 80613588 | *RAB40B* | 0·48 | 2·94E-04 |
| rs7098190 | 10 | 125457952 | *3.8kb3' of GPR26* | 0·30 | 2·98E-04 |
| rs10281505 | 7 | 20716244 | *ABCB5* | 0·18 | 2·99E-04 |
| rs2577254 | 2 | 11975372 | *1.7kb3' of MIR4262* | 0·47 | 3·19E-04 |
| rs7672688 | 4 | 113001776 | *7.9kb5' of RP11-269F21.3* | 0·46 | 3·23E-04 |
| rs11980286 | 7 | 50043518 | *ZPBP* | 0·17 | 3·40E-04 |
| rs2913366 | 5 | 5417809 | *3kb5' of KIAA0947* | 0·46 | 3·56E-04 |
| rs6835978 | 4 | 47500814 | *ATP10D* | 0·24 | 3·69E-04 |
| rs1225132 | 11 | 76551635 | *20kb5' of ACER3* | 0·20 | 3·77E-04 |
| rs7278106 | 21 | 21075420 | *190kb3' of AP000946.2* | 0·39 | 3·78E-04 |
| rs11236557 | 11 | 75552129 | *UVRAG* | 0·34 | 3·87E-04 |
| rs1371121 | 7 | 149375086 | *37kb5' of KRBA1* | 0·27 | 4·07E-04 |
| rs1034331 | 21 | 21141056 | *125kb3' of AP000946.2* | 0·39 | 4·39E-04 |
| rs6833846 | 4 | 113924858 | *RP11-650J17.1* | 0·22 | 4·43E-04 |
| rs7946600 | 11 | 99925167 | *CNTN5* | 0·28 | 4·44E-04 |
| rs13232564 | 7 | 18952433 | *HDAC9* | 0·16 | 4·76E-04 |
| rs2407254 | 21 | 21071070 | *195kb3' of AP000946.2* | 0·31 | 4·81E-04 |

| **Supplementary table 2. Clinicopathological features of 149 stage I-II NSCLC patients** | |
| --- | --- |
| **Patient characteristics** | **n (%)** |
| **Age (years)** |  |
| ≤65 years | 74 (49·7) |
| >65 years | 75 (50·3) |
| **Gender** |  |
| Male | 119 (79·9) |
| Female | 30 (20·1) |
| **Histology** |  |
| ADC | 73 (49·0) |
| SCC | 59 (39·6) |
| Other | 17 (11·4) |
| **Stage** |  |
| I | 101 (67·8) |
| II | 48 (32·2) |
| **Smoking history** |  |
| Never | 15 (10·1) |
| Former | 101 (67·8) |
| Current | 33 (22·1) |
| **Treatment** |  |
| Surgery | 36 (24) |
| Surgery and adjuvant chemotherapy | 113 (76) |

ADC: adenocarcinoma; SCC: squamous cell carcinoma.
