# Supplementary material for: Genomic characterization of individuals presenting extreme phenotypes of high and low risk to develop tobacco‐induced lung cancer
Source: Cancer Med. 2018 May 15;7(7):3474–83. doi: 10.1002/cam4.1500 (PMC6051154; doi:10.1002/cam4.1500)
Supplement: Supplementary file 2 — Appendix S1. Immunohistochemical analysis. [file CAM4-7-3474-s002.docx]

**Supplementary Appendix S1**: Immunohistochemical analysis.

For PDE10A immunohistochemical assay, paraffin removal, endogenous peroxidase activity quenching and antigen retrieval were performed as previously described (Grbesa *et al*, 2015). Subsequently, sections were incubated in a humidity chamber overnight at 4ºC with an anti-human PDE10A antibody (Genetex) diluted 1:500 in REAL antibody diluent (Dako). After applying the Envision polymer (Dako) for 30 minutes at room temperature, sections were developed with diaminobenzidine and counterstained with hematoxylin. The specificity of PDE10A antibody was demonstrated by Western Blot analysis and immunocytochemistry of cell lines expressing different levels of the protein. Also isotype and negative (omission of the primary antibody) controls were performed. Staining scores were established by semiquantitative analysis. Briefly, the extension (percentage of positive cells; 0-100%) and the intensity of the staining (1, mild; 2, moderate; and 3, intense labeling) was evaluated independently by two observers. A final score was calculated by adding the products of the percentage cells stained at a given staining intensity and the staining intensity. The median value was chosen as the cutoff point to separate low from high PDE10A expressing tumors. Discordant independent readings were resolved by simultaneous review by the two observers.

**References.**

Grbesa I, Pajares MJ, Martínez-Terroba E, Agorreta J, Mikecin A-M, Larráyoz M, Idoate MA, Gall-Troselj K, Pio R, Montuenga LM (2015) Expression of sirtuin 1 and 2 is associated with poor prognosis in non-small cell lung cancer patients. *PLoS One* **10**: e0124670, doi:10.1371/journal.pone.0124670.
